# Plant species, metabolites, and environmental factors shape the phyllosphere microbiota of grazed grasslands

**DOI:** 10.1128/aem.01289-25

**Published:** 2026-01-21

**Authors:** Marion Dalmasso, Annette Morvan-Bertrand, Didier Goux, Nicolas Elie, André Sesboüé, Frédéric Meuriot, Caroline Chagnot, Margot Schlusselhuber, Nathalie Desmasures, Frédéric Launay, Nathalie Noiraud-Romy, Marie-Pascale Prud'homme, Marina Crétenet

**Affiliations:** 1 Université de Caen Normandie, Normandie Univ, ABTE UR 4651 27003, Caen, France; 2 Université de Caen Normandie, INRAE, Normandie Univ, EVA UMR 950 27003, Caen, France; 3 Université de Caen Normandie, Normandie Univ, US EMerode, CMAbio3 27003, Caen, France; 4 Université de Caen Normandie, CNRS, Normandie Univ, LMNO UMR 6139 27003, Caen, France; 5 INRAE, Unité Expérimentale du Pin, Le Pin-au-Haras, France; 6 Université de Caen Normandie, Normandie Univ, US EMerode, CBM 27003, Caen, France; The University of Tennessee Knoxville, Knoxville, Tennessee, USA

**Keywords:** grassland, microbiome, plant carbohydrates, microbiota, phyllosphere

## Abstract

The phyllosphere of permanent grasslands is a reservoir of microbial diversity, yet its composition and the factors influencing its fluctuations are still poorly understood. The aim of this study was to identify the factors driving the structure of the phyllosphere microbiota. Three plant species of two adjacent grasslands, one of the two receiving nitrogen (N) fertilization, were selected: two fructan-accumulating grasses (perennial ryegrass, *Lolium perenne* and Yorkshire fog, *Holcus lanatus*) and a non-fructan species (white clover, *Trifolium repens*). Samples were collected at three periods—two in summer and autumn—and the weather parameters were recorded. Bacterial enumerations and metabarcoding analysis of the plant microbiota were performed. Carbon and N) contents of the leaves and stomatal conductance regulation were assessed. The composition of leaf microbiota was determined mainly by the plant host species*—T. repens* grouping separately from the other plant species—, the period of the year (early summer versus late summer and autumn), and, to a lesser extent, fertilization. *Methylobacterium* was particularly dominant in *T. repens*. A significant effect of pinitol and fructans levels on the composition of the phyllosphere microbiota was observed in *T. repens* and in the *Poaceae* species, respectively. The daily temperature range, the rainfall amount, the daily average radiation, and evapotranspiration were significant drivers of the phyllosphere microbiota composition. This study provides insights into plant and environmental factors that shape grassland phyllosphere microbiota.

**IMPORTANCE:**

The phyllosphere is estimated to cover more than 10^8^ km^2^ over the globe, and grasslands cover about 30%–40% of the world land area. Grasslands host a vast microbial reservoir, characterized by both the abundance and diversity of microorganisms. Evidence of connections between phyllosphere and raw milk microbiota exists. The significance of our research is in highlighting grasslands as potential reservoirs of beneficial microorganisms for plants and dairy-derived products. The phyllosphere microbiota should be considered when evaluating the ecosystem services of grasslands as it contributes to supporting services by enhancing biodiversity and to provisioning services through microorganisms that can benefit agriculture and the food industry.

## INTRODUCTION

The terrestrial leaf surface (phyllosphere) is estimated to represent over 10^8^ km^2^ over the globe and is colonized by bacteria, filamentous fungi, yeasts, algae and less frequently by protozoa and nematodes (1). Bacteria are often found in numbers averaging 10^4^ to 10^5^ cells/mm^2^ of the leaf surface (up to 10^8^ cells/g of leaf), outnumbering the cells of the plants themselves (1, 2). Grasslands cover 30%–40% of the world land area (3). Due to the remarkable plant diversity in certain grasslands, particularly permanent grasslands, their phyllosphere represents a vast reservoir of microbial diversity. Leaf-associated microbial communities are host-specific and shaped by plant and environmental factors, yet the determinants of their structure in grassland ecosystems remain poorly understood. Previous studies have either characterized grassland microbiota at the community level or examined individual species in controlled settings, but little is known about their assembly in grazed permanent grasslands (4–9). Regarding phyllosphere bacterial communities, an increasing amount of data is available, covering trees (10–13) and herbaceous species (14–19). More recently, a few studies focused on the leaf microbiota of some grassland species (3, 8, 20–23). A comparison between nine grassland species, including *Poaceae*, *Fabaceae,* and other eudicotyledons, revealed a rich phyllosphere microbiota dominated by *Pseudomonadota* among bacteria with most operational taxonomic units (OTUs) being detected across different species (22).

Much of cow milk production in temperate regions originates from grazed grasslands. Recent studies suggest that microorganisms from the soil and phyllosphere microbial communities of these grasslands are transferred to raw milk, either through contact with cow teats during milking or via forage-based feeding. An evaluation of microbial transfers in dairy farms of the Comté cheese area highlighted that 37% to 47% of the identified genera were shared from soil to milk via the phyllosphere and teats. A link between the phyllosphere and raw milk microbiota was especially demonstrated using a consensus network analysis (9). Doyle et al. (24) also reported that raw milk microbial communities were more similar to those found in grass leaves and soil when cows grazed, whereas they resembled the microbiota of silage when cows were fed indoors. A greater relative abundance of environmental bacteria such as *Corynebacterium, Pseudomonas, Acinetobacter,* and *Lactococcus* was found in raw milk of grazing cows (24). Leaf samples collected in grazed grasslands in a dairy environment by Falardeau et al. (25) were dominated by *Pseudomonadota*, including the orders *Pseudomonadales*, *Enterobacterales*, *Burkholderiales,* and *Rhizobiales*. The orders *Flavobacteriales* and *Actinomycetales* were more abundant in samples collected in grazed grasslands than stored hay, whereas it was the contrary for *Enterobacterales* (25).

The adaptation of the microbial communities to live in the phyllosphere depends on a variety of mechanisms related to a diversity of physicochemical and biotic constraints (26). Climatic factors, including temperature and seasonal changes, as well as agricultural practices such as pesticide application and fertilization, play a crucial role in shaping the community structure (26). Climate change is expected to reshape phyllosphere microbial communities, with implications for pathogen transmission in agro-ecosystems (3). Understanding how plant diversity, particularly in species-rich grasslands promoted by agroecological practices, influences microbiota diversity is therefore crucial (27). Epiphytic microbial colonization depends on the availability of sugars, mainly sucrose, glucose, and fructose, as a carbon source, which is limited by sugar leaking from the apoplasts and by their replenishment rate from the internal leaf (28, 29). The relationship between sugar availability and bacterial colonization varied significantly among plant species (28), suggesting that colonization may be influenced not only by sugar composition but also by the availability of other mineral or organic nutrients. The availability of volatile organic compounds depends on the stomatal opening, which is influenced by plant physiology and morphology and environmental conditions (30). Regarding soluble carbohydrates, plant leaves may accumulate not only sucrose, glucose, and fructose but also polyols, such as mannitol, sorbitol, pinitol (31), galactosyl- and fructosyl-oligosaccharides and fructans (fructosyl-polysaccharides) (32, 33). Their amount and composition inside the leaf hugely vary among plant species and environmental conditions (34), which may alter soluble carbohydrate availability and profiles on the leaf surface (35). Moreover, leaf soluble carbohydrates may modulate the structure of microbiota not only due to their role as a carbon source but also due to their direct involvement as signaling molecules in plant-microorganism interactions (36). Few studies have investigated the relationship between phyllosphere microbial community composition and leaf biochemical composition, with most focusing on the role of plant secondary metabolites such as glucosinolates and carotenoids (37) and essential oils (38). To our knowledge, no data are currently available on the relationship between leaf carbohydrate profiles and the structures of microbial communities.

The impacts of leaf biochemical composition, which varies with plant host species and seasonal weather conditions, on the leaf bacterial community diversity remain poorly understood. Therefore, the current study aimed to investigate the structure of the phyllosphere microbiota of three dominant plant species in grazed temperate grasslands and to identify the key factors driving the structure of the phyllosphere microbiota, which included plant internal factors (leaf biochemical composition) and external factors (fertilization level, season, and weather conditions). Given the role of soluble carbohydrates in microbial nutrition and plant-microorganism interactions, we hypothesized that the soluble carbohydrate composition of plant species contributes to shape the structure of bacterial communities. To assess this hypothesis, we selected three species among the dominant plant species of the studied temperate grasslands, two fructan-accumulating grasses (perennial ryegrass, *Lolium perenne* and Yorkshire fog, *Holcus lanatus*) and a non-fructan species from the nitrogen-fixing *Fabaceae* family that accumulates pinitol (white clover, *Trifolium repens*). The three species can also be distinguished by the epidermis of their leaves, which is hairless for *L. perenne* and *T. repens* and covered with soft hairs for *H. lanatus*. To compare the effect of soluble carbohydrate composition among other internal factors, we also analyzed the relationships between microbial communities and carbon (C) and nitrogen (N) contents of the leaves, as well as with δ^13^C, used here as an indicator of the stomatal conductance regulation, with a low δ^13^C indicating a higher ^13^C discrimination due to higher conductance (39). To capture a wide range of variations in the biochemical composition (internal factors), leaves were sampled at three different periods (early summer, late summer, and autumn), on two adjacent grazed grasslands sharing the same topology, soil composition, and cattle grazing management but differing in their level of N fertilization. As leaves were sampled at different dates along the growing period in the two conditions of fertilization, the effects of corresponding external factors (sampling period and fertilization level) and weather parameters possibly influencing the plant microbiota structure were also investigated.

## RESULTS

### Microbial enumeration and localization at the leaf surface

**Fig 1:**
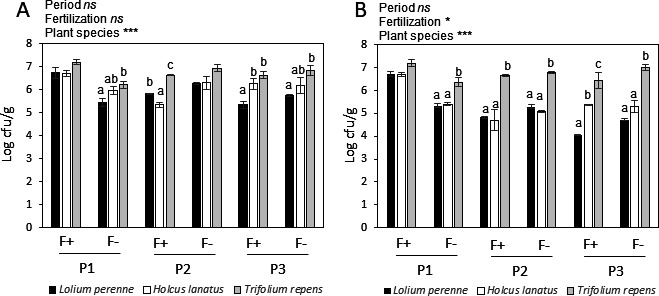
Microbial counts for each plant species *Lolium perenne*, *Holcus lanatus*, and *Trifolium repens*. (**A**) Standard plate counts; (**B**) Methylobacteria. F+: grassland receiving fertilization, F−: grassland not receiving any fertilization. P1: early summer, P2: late summer, and P3: autumn. Bacterial counts were expressed as mean values of three replicates in colony-forming units per gram of leaf (cfu/g). The effects of the period, fertilization level, and plant species are shown for each panel (ns: not significant, *P < 0.05, **P < 0.01, and ***P < 0.001). Inside each period and fertilization level, letters indicate significant differences between plant species (*P* < 0.05).

**Fig 2:**
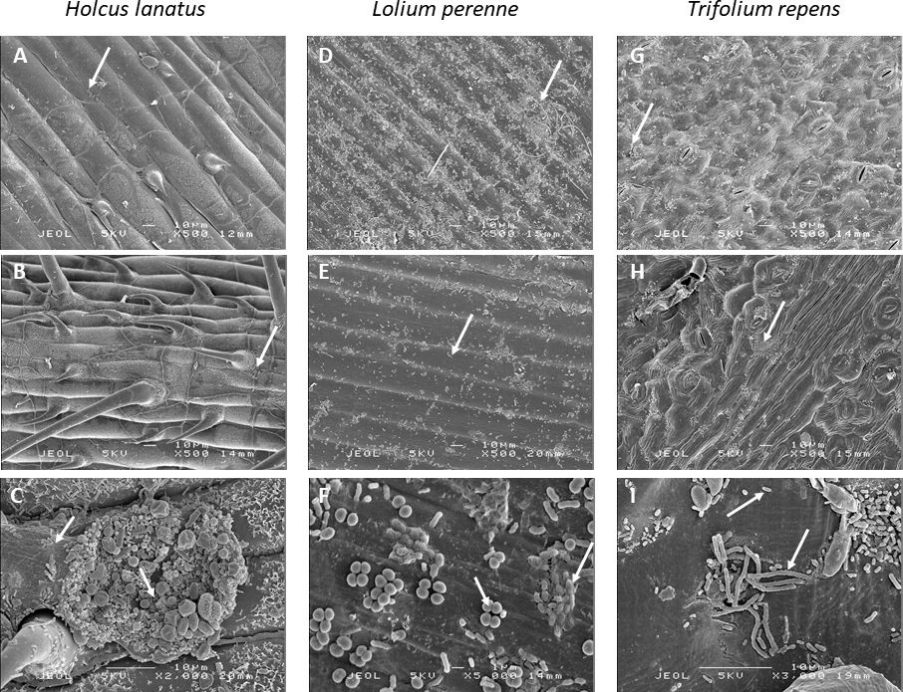
Scanning electron micrographs illustrating the diversity of microorganisms colonizing the abaxial leaf surface of *Holcus lanatus* (**A–C**), *Lolium perenne* (**D–F**), and *Trifolium repens* (**G–I**). Arrows indicate microorganisms that were visible at the leaf surface.

To evaluate the structure of the phyllosphere microbiota found in grasslands, we sampled three plant species leaves (*L. perenne*, *H. lanatus*, and *T. repens*) at three sites in two grasslands, one receiving fertilization (F+) and one with no fertilization (F−), at three sampling periods (early summer, P1; late summer, P2; and autumn, P3). Overall, there was no effect of the sampling period and fertilization on the total microbial population grown on plate count agar (PCA) (Fig. 1A). The plant species effect (*P* < 0.001) was mainly due to higher levels of bacteria found on the leaves of the *Fabaceae T. repens* than on the leaves of the grasses *L. perenne* and *H. lanatus*. Presumed *Methylobacterium* population levels on *Methylobacterium* agar were also significantly higher (*P* < 0.05) in *T. repens* than in *L. perenne* and *H. lanatus* samples, especially at P2 and P3 (Fig. 1B). A slight effect of fertilization was observed on the presumed *Methylobacterium* population density, but this varied according to the season. Density was increased by fertilization in P1 and decreased in P3 (Fig. 1B). The scanning electron micrographs revealed a great epiphytic microbial diversity at the abaxial leaf surface of *H. lanatus* (Fig. 2A–C), *L. perenne* (Fig. 2D–F), and *T. repens* (Fig. 2G–I). Microorganisms were present with a large diversity of forms and length of cells, some being rod-shaped bacillus-like cells and others coccus-like cells (Fig. 2F and I). They were also found as single cells (Fig. 2E) and forming aggregates (Fig. 2C and H).

### Composition and structure of the phyllosphere microbiota

A 16S rRNA gene-based metabarcoding analysis was performed in order to investigate the phyllosphere microbiota composition and structure of *H. lanatus*, *L. perenne,* and *T. repens*. The data set contained 1,122 OTUs with 208,866 to 1,110,755 sequences per sample for a total of 42,558,585 quality sequences from 54 samples, after clustering, chimera removal, and filtering. The alpha diversity indexes—observed species, Chao1, Shannon, and InvSimpson—were represented in the form of box plots for *H. lanatus*, *L. perenne,* and *T. repens* (see Fig. S1 at https://doi.org/10.57745/MDJY8I). Observed species and Chao1 indexes were significantly higher for *H. lanatus* than for the other two plants (*P* < 0.001), indicating a more diverse bacterial community in *H. lanatus* than in *L. perenne* and *T. repens* (see Fig. S1A and B at https://doi.org/10.57745/MDJY8I). *L. perenne* bacterial community was significantly the less diverse of the three plant species (*P* < 0.001). Shannon and InvSimpson indexes were significantly higher for *L. perenne* and *H. lanatus* than for *T. repens* (*P* < 0.001), which indicated that the microbiota of *L. perenne* and *H. lanatus* was not marked by dominating OTUs contrary to *T. repens* microbiota (Fig. S1C and D).

**Fig 3:**
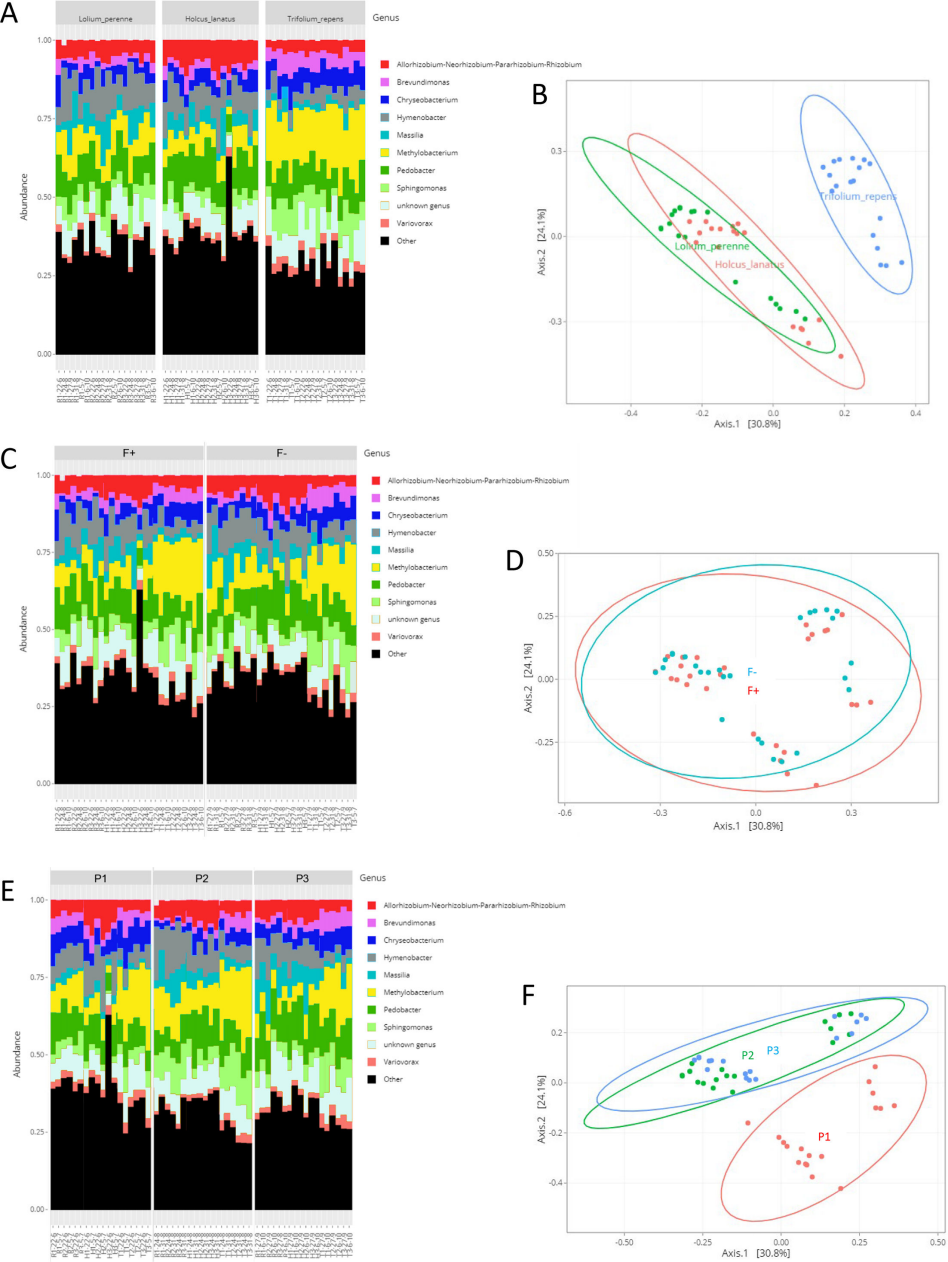
Microbiota composition (10 top genera) and Bray-Curtis PCoA plots of the phyllosphere of the three plants (**A and B**, respectively), in the two grasslands (**C and D**, respectively), and at the three sampling periods (**E and F**, respectively). Fertilization, F+: grassland receiving fertilization; F−: unfertilized grassland; Period, P1: early summer; P2: late summer; P3: autumn; Samples are named as follows: Plant species (H: *H. lanatus*, R: *L. perenne*, T: *T. repens*)_Sampling zone number (1, 2, 3)_Sampling date.

The phyllosphere bacterial community composition varied among plant species (Fig. 3A). This was corroborated by a principal coordinate analysis (PCoA) based on Bray-Curtis distances to explore the structure of the phyllosphere microbiota showing that *T. repens* samples form a distinct group from *L. perenne* and *H. lanatus* samples (Fig. 3B). The phyllosphere microbiota composition and structure did not seem to vary according to fertilization (F+ and F−) (Fig. 3C and D). Even if no differences in the major genera composition of the different sampling periods (P1, P2, and P3) seemed to appear (Fig. 3E), the PCoA analyses indicated that the samples from P1 grouped separately from the samples collected on P2 and P3 (Fig. 3F), indicating a microbiota structure specific to P1 compared to P2 and P3.

The proportion of unknown genera in all the samples represented 3.8% to 15.7% of the sequences, depending on the sample (Fig. 3). The phyllosphere bacterial communities of *T. repens* samples were dominated by *Methylobacterium*, where it represented 11.7% to 27.8% of the sequences (Fig. 3A). *Sphingomonas* and *Pedobacter* were the second- and third-most abundant genera in *T. repens* microbiota, accounting for up to 17.5% and 13.1% of the sample sequences, respectively. The bacterial microbiota composition of *L. perenne* and *H. lanatus* was noticeably the same, with *Hymenobacter*, *Methylobacterium,* and *Pedobacter* genera each adding up 4% to 19% of the sequences depending on the sample. *Rhizobium* was more abundant in *H. lanatus* microbiota (5.5% to 12.9% of the sequences) than in *L. perenne* (3.5% to 7.5%), whereas *Sphingomonas* was more abundant in *L. perenne* (2.7% to 11%) than in *H. lanatus* (2% to 5.7%) (Fig. 3A). These results were confirmed by the differential analysis of OTUs at the genus level in the samples. When comparing *T. repens* with *H. lanatus* samples, 100 genera showed variations, including 40 genera more present (*P* < 0.01, Log2FoldChange > 0) and 60 genera less present (*P* < 0.01, Log2FoldChange < 0) in *T. repens* than in *H. lanatus* (see Fig. S2A at https://doi.org/10.57745/MDJY8I). *Methylobacterium*, *Brevundimonas*, *Chryseobacterium*, *Dyadobacter*, *Variovorax*, *Sphingobium*, *Sphingomonas,* and *Rhodococcus* were especially significantly more present in *T. repens* (*P* < 0.01) than in *H. lanatus*, whereas *Frigoribacterium*, *Rhizobium*, *Massilia*, *Mucilaginibacter,* and *Xylophilus* were significantly less present in *T. repens* than in *H. lanatus* (*P* < 0.01). *Hymenobacter* and *Pedobacter* had a clear tendency to be less present in *T. repens* than in *H. lanatus* (see Fig. S2A at https://doi.org/10.57745/MDJY8I). Almost the same tendencies in genera variations were observed when comparing *T. repens* to *L. perenne* (see Fig. S2B at https://doi.org/10.57745/MDJY8I), with 104 genera that varied, including 49 genera significantly more present (*P* < 0.01, Log2FoldChange > 0) and 55 genera significantly less present (*P* < 0.01, Log2FoldChange < 0) in *T. repens* than in *L. perenne*. The most noticeable difference from the previous comparisons was the significant prevalence of *Rhizobium* (*P* < 0.01) in *T. repens* compared to *L. perenne* (see Fig. S2B at https://doi.org/10.57745/MDJY8I). The prevalence of only 72 genera significantly varied (*P* < 0.01) between *L. perenne* and *H. lanatus*, with 27 more present and 45 less present in *L. perenne* than in *H. lanatus. Frigoribacterium*, *Pantoea*, *Methylobacterium,* and *Hymenobacter* were especially more present in *L. perenne* than in *H. lanatus*, whereas it was the contrary for *Pedobacter*, *Luteibacter*, *Mucilaginibacter*, *Aeromicrobium,* and *Rhodopseudomonas* (see Fig. S2C at https://doi.org/10.57745/MDJY8I).

### Identification of the factors driving the phyllosphere microbiota structure and composition

The relative influences of the plant species, the sampling date, the fertilization level, and of the interactions between those factors on the phyllosphere community structure were quantified by conducting a permutational multivariate analysis of variance (PERMANOVA) (40) on Bray-Curtis dissimilarities among samples (Table 1). The strongest drivers of the phyllosphere microbiota structure were the plant species (R² = 32%, *P* < 0.001) and the sampling date (R² = 26.7%, *P* < 0.001). Fertilization had a weaker effect (R² = 2.7%, *P* < 0.001) than the other factors but was still a significant driver of phyllosphere microbiota structure. All second-level interactions were significant, the strongest being the interaction between plant species and sampling date (R² = 11%, *P* < 0.001) (Table 1). The third-level interaction was not significant (R² = 2%, *P* = 0.42). The same results were obtained when performing the same analysis with the distance square roots to minimize the influence of abundant OTUs (data not shown). This revealed that not only the abundant OTUs but also the whole phyllosphere microbiota structure was driven by plant species, date of sampling, and the interaction of these factors.

**TABLE 1:** Bacterial community structure explained by sampling period, plant species, and fertilization and their interactions^*a*^

Variable	Bray-Curtis dissimilarity		
	F value	R²	Pr(>F)
First level			
Plant species	31.92	0.32	0.001
Period	26.73	0.27	0.001
Fertilization	5.30	0.027	0.001
Second level			
Period × plant species	5.73	0.11	0.001
Period × fertilization	3.54	0.036	0.001
Plant species × fertilization	2.64	0.027	0.004
Third level			
Period × plant species × fertilization	1.04	0.02	0.418

^*a*^ Data represent results of PERMANOVA analysis of Bray-Curtis dissimilarities. The model explained 81.8% of the variation in bacterial taxonomical and phylogenetic community structure. Pr(>F): *P* value. ×: interactions between variables.

**Fig 4:**
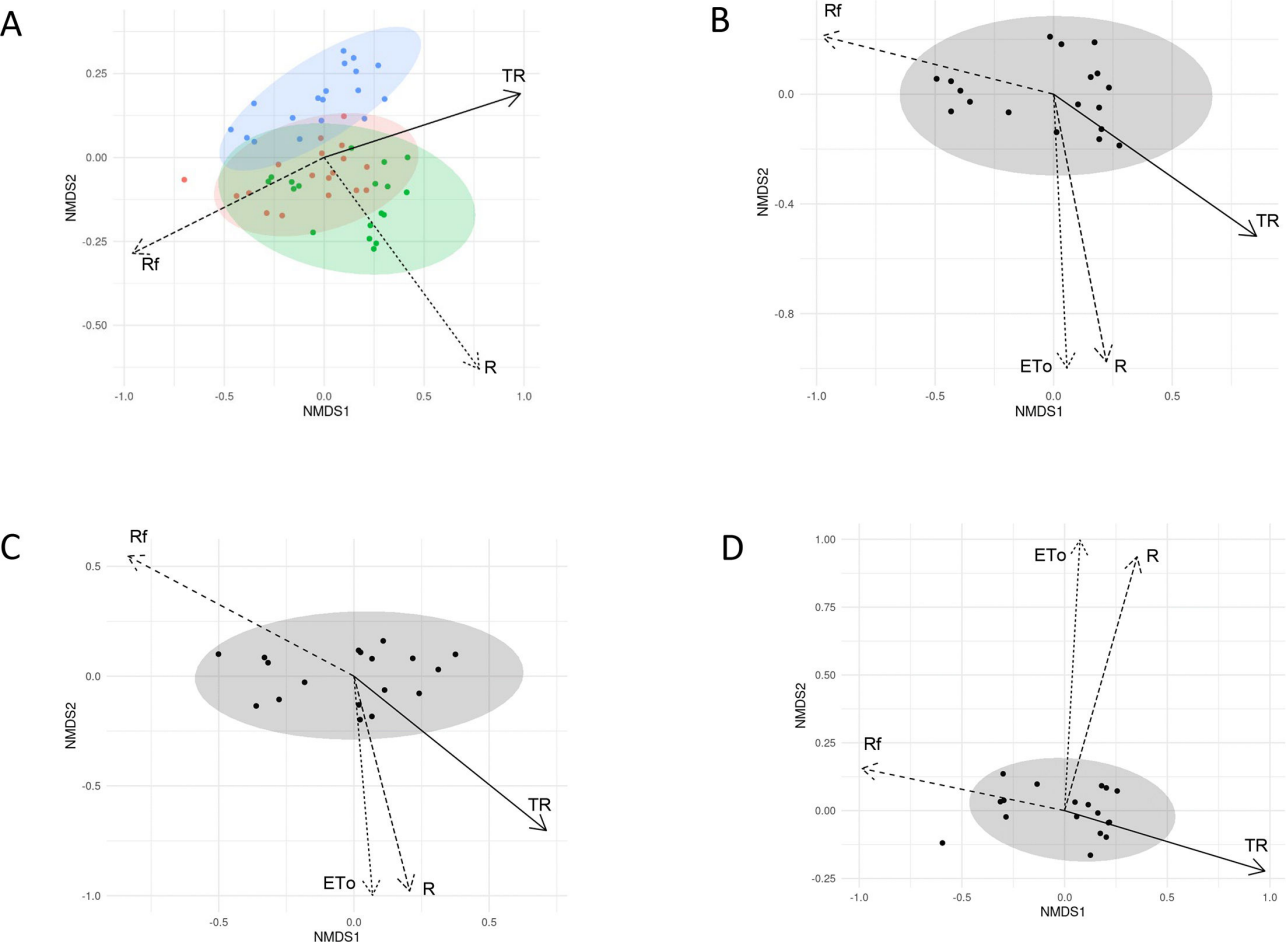
NMDS ordination of variation in the bacterial community structure of plant species before sampling, based on Bray-Curtis distances, in relation with weather variables at 3d. (**A**) Samples (points) are colored based on the plant species (green for *L. perenne*, red for *H. lanatus*, and blue for *T. repens*); (**B**) NMDS for *L. perenne* samples; (**C**) NMDS for *T. repens* samples; (**D**) NMDS for *H. lanatus* samples. For all panels, arrows represent the significant correlation on the NMDS axis between weather factors at 3d before sampling versus the relative abundance of OTUs in communities. ETo: daily Penman-Monteith evapotranspiration; R: daily average radiation; Rf: rainfall; TR: daily temperature range.

To further explain the differences in phyllosphere microbiota between plant species, we also examined the influence of external factors related to weather conditions and internal factors related to plant physiology and biochemistry. Regarding external factors, meteorological parameters such as rainfall (Rf), daily temperature range (TR), Daily Penman-Monteith evapotranspiration (ETo), daily average radiations (R), and daily average wind speed (W) were collected. The mean values of each parameter recorded on the 3 days (3d) and the 15 days (15d) before each sampling date were used. The phyllosphere microbiota structure patterns for the plant species, together and alone, according to these weather factors were illustrated by performing nonmetric multidimensional scaling (NMDS) ordinations of Bray-Curtis dissimilarities (Fig. 4), which generated acceptable stress values below 0.15 (41). The phyllosphere microbiota structure in all samples was significantly driven by TR (*P* < 0.001), Rf (*P* < 0.001), R (*P* < 0.005), and ETo (*P* < 0.042) at 3d (Fig. 4A; see Table S1 at https://doi.org/10.57745/MDJY8I). Rf_3d and TR_3d had the highest influence (R² = 63.6% and R² = 58.1%, respectively; see Table S1 at https://doi.org/10.57745/MDJY8I) with opposite orientations on the first NMDS axis (Fig. 4A). R and ETo had a weaker influence (R² = 19.5% and R² = 10.8%, respectively; see Table S1 at https://doi.org/10.57745/MDJY8I) with joint orientations on the second NMDS axis (Fig. 4A). These trends were also true when considering *L. perenne* (Fig. 4B), *T. repens* (Fig. 4C), and *H. lanatus* (Fig. 4D) samples alone (see Table S1 at https://doi.org/10.57745/MDJY8I). The only difference was that R and ETo vectors pointed up along the second NMDS axis for *H. lanatus,* whereas they pointed down the second NMDS axis for the other plant species (Fig. 4B through D). The same weather factors appeared to have a significant influence on the phyllosphere microbiota of the three plant species considering the previous 15 days (15d), with the apparition of wind speed as another influencing factor (see Fig. S3 and Table S1 at https://doi.org/10.57745/MDJY8I).

**Fig 5:**
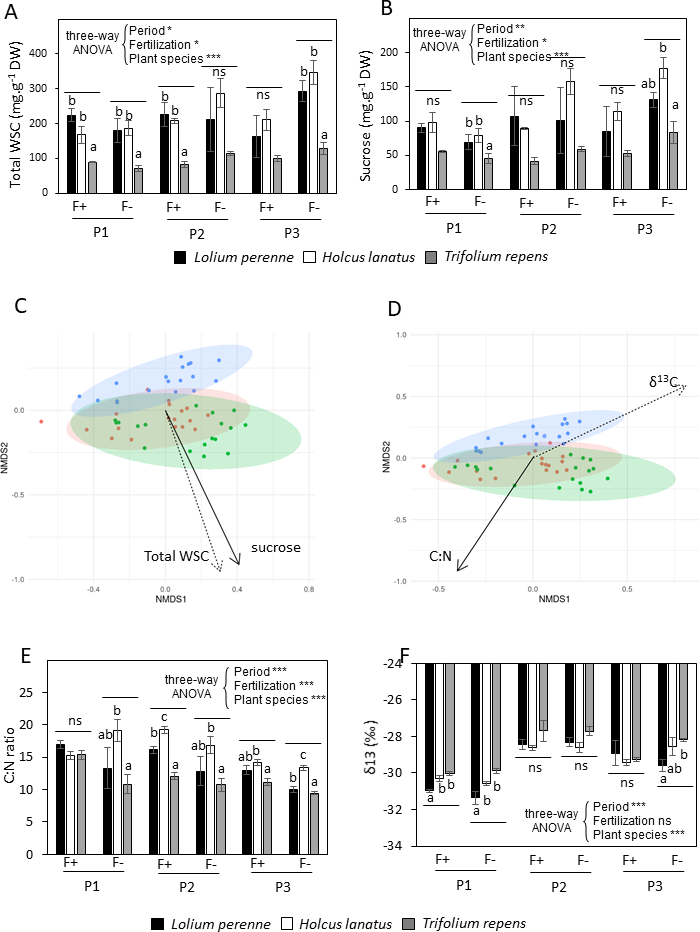
Leaf composition in total WSC, sucrose, C:N ratio, and δ^13^C and structure of the bacterial population. (**A**) Total WSC concentration in leaves of the three plant species; (**B**) Sucrose concentration in leaves of the three plant species; (**C**) NMDS ordination of variation in the bacterial community structure of plant species before sampling based on Bray-Curtis distances in relation with total WSC and sucrose concentrations; (**D**) NMDS ordination of variation in the bacterial community structure of plant species before sampling based on Bray-Curtis distances in relation with C:N ratio and δ^13^C in leaves. Samples (points) are colored based on the plant species (green for *L. perenne*, red for *H. lanatus*, and blue for *T. repens*); (**E**) C:N ratio in leaves of the three plant species; (**F**) δ^13^C in leaves of the three plant species. Fertilization, F+: grassland receiving fertilization, F−: unfertilized grassland. Periods, P1: early summer, P2: late summer, P3: autumn. WSC concentration was expressed as mean values ± standard error of three replicates (mg.g^−1^ DW). The effects of period, fertilization level, and species were analyzed by three-way analysis of variance (ANOVA) and shown in each panel (*ns* not significant, *P < 0.05, **P < 0.01, and ***P < 0.001). Inside each period and fertilization level, a one-way ANOVA followed by Tukey’s test was used to determine significant differences between species (*P* < 0.05), which are indicated by different letters.

Regarding internal factors, the leaf total water-soluble carbohydrate (WSC) composition, the C:N ratio, and the δ13C (assessed by ^13^C isotopic signature) were considered (Fig. 5; see Fig. S4 at https://doi.org/10.57745/MDJY8I). For each of the three sampling periods, the total level of WSC was much lower in the leaves of clover (*T. repens;* around 100 mg.g^−1^dry weight [DW]) than in the leaves of the two grass species (*L. perenne* and *H. lanatus*; between 200 and 350 mg.g^−1^DW) (Fig. 5A). The difference between clover and the two grasses was mainly due to the accumulation of fructans in the grasses (see Fig. S4A at https://doi.org/10.57745/MDJY8I). The main WSCs were fructans and sucrose (both between 100 and 200 mg.g^-1^ DW) in the grasses, and sucrose (around 50 mg.g^-1^ DW) in clover (Fig. 5B). The leaves of clover contained high levels (up to 40 mg.g^-1^ DW) of pinitol, an *O*-methylated cyclitol common in *Fabaceae* (see Fig. S4B at https://doi.org/10.57745/MDJY8I). The total WSC level was slightly affected by the sampling period and the level of fertilization, with the highest content found in the leaves of grasses sampled in the grassland not receiving fertilization (F−) during P3, attributed to the accumulation of sucrose and fructans (Fig. 5A and C; see Fig. S4A at https://doi.org/10.57745/MDJY8I).

The phyllosphere microbiota structure patterns according to internal factors have been illustrated by NMDS ordinations for plant species taken together or alone (Fig. 5C and D; see Table S1 at https://doi.org/10.57745/MDJY8I). When the plant species were taken together, the microbiota structure was significantly driven by the levels of total WSC (*P* = 0.002; R^2^ = 25.6%) and sucrose (*P* = 0.023; R^2^ = 14.2%) with similar orientations on the negative values of the second NMDS axis that distinguished the clover from the two grasses (Fig. 5C). Glucose and fructose contents had no significant influence on the microbiota structure (see Table S1 at https://doi.org/10.57745/MDJY8I). Fructans and pinitol were excluded from the NMDS analysis when the three plant species were considered altogether since these two compounds did not accumulate in clover and grasses, respectively. When *T. repens* was considered alone, the phyllosphere microbiota structure was significantly driven by the contents of pinitol (*P* = 0.001; R^2^ = 68.6%) and sucrose (*P* = 0.006; R^2^ = 4.2%), with a positive correlation with the first and second axes, respectively (see Table S1 at https://doi.org/10.57745/MDJY8I). When *L. perenne* and *H. lanatus* were considered alone, no significant influence of any WSC (*P* > 0.05) was observed. A slight influence (*P* = 0.052; R^2^ = 30.5%) was observed for sucrose in *H. lanatus* (see Table S1 at https://doi.org/10.57745/MDJY8I).

The C:N ratio of the leaves, used as an indicator of C and N metabolism, varied considerably between samples, from less than 10 to almost 20 (Fig. 5E). It was generally the highest in *H. lanatus* and the lowest in *T. repens* (Fig. 5E), mainly due to low and high contents of N, respectively (see Fig. S4F at https://doi.org/10.57745/MDJY8I). The C:N ratio was significantly modified by the sampling period and fertilization level (Fig. 5E). It decreased in each plant species throughout the sampling period, mainly due to an increase in the N content (see Fig. S4F at https://doi.org/10.57745/MDJY8I). At the same time, the fertilization slightly increased the C:N ratio (Fig. 5E) by reducing N dilution (see Fig. S4F at https://doi.org/10.57745/MDJY8I). Carbon isotopic signature (δ^13^C) was used as an indicator of stomatal conductance regulation; a low δ^13^C value indicates a high ^13^C discrimination due to high stomatal conductance. δ^13^C appeared to depend on the plant species and the sampling period (Fig. 5F). It was generally the lowest in *L. perenne* and the highest in *T. repens*, indicating higher stomatal conductance in *L. perenne* (Fig. 5F). It increased throughout the sampling period (Fig. 5F), revealing a decrease in stomatal conductance. When the plant species were taken together, the microbiota structure was significantly driven by δ^13^C (*P* = 0.001; R^2^ = 53.5%) and to a slightly lesser extent by the C:N ratio (*P* = 0.001; R^2^ = 41.5%) with opposite directions in the first plan of the NMDS (Fig. 5D; see Table S1 at https://doi.org/10.57745/MDJY8I). When *L. perenne* and *T. repens* were considered alone, the same effects were observed, while the microbiota structure was only significantly influenced by δ^13^C in *H. lanatus* (see Table S1 at https://doi.org/10.57745/MDJY8I).

## DISCUSSION

In this study, we describe the phyllosphere bacterial communities of three plant species sampled from two grasslands, differing by their fertilization level, at different periods of the year. We showed that the composition of plant leaf bacterial communities of grazed grasslands is determined mainly by the plant host species and the period of the year (P1 versus P2 and P3), and to a lesser extent to fertilization of the grassland.

### Microbiota composition and microbial transfers

In our study, counts of cultivable microorganisms were correlated to the plant species but not to sampling times and fertilization. The number of total bacteria is often underestimated by a culture-dependent method as shown by Rastogi et al. (42) with a reduction of 1 to 3 orders of magnitudes compared to quantitative PCR (42). Cultivable bacteria (e.g. plate counts [CFU]) constitute from 0.1% to 35% of total bacteria (43). In addition, metabarcoding data showed that the bacterial composition also differed between plant species, with *T. repens* microbiota being grouped separately from those of *L. perenne* and *H. lanatus* and having a higher diversity.

The plant phyllosphere has been found to be predominantly colonized by strict aerobes, especially represented by *Methylobacteriaceae* and *Sphingomonadaceae* (*Alphaproteobacteria*) (44), which was confirmed in the present study. *Methylobacterium* was particularly dominant in *T. repens*. This genus has particular fitness traits that make it more resistant to external threats. For instance, *Methylobacterium* harbors a UVA-absorbing compound, with absorbing strength and spectrum close to those of avobenzone, which could explain its aptitude to colonize plant leaves (45).

*Methylobacterium, Sphingomonas,* and *Variovorax* were three of the top ten genera in this study, which is consistent with the fact they are three out of the five genera used when building phyllosphere synthetic communities (15). In grasslands, the phyllosphere was also characterized by high relative abundance of *Pseudomonadota* (e.g. *Methylobacterium, Massilia, Sphingomonas, Variovorax, and Pseudomonas*) and thus confirmed our results (9, 25). Special attention should be given to *Actinomycetota*, *Bacteroidota*, and *Bacillota*, which are also identified as core members of the phyllosphere community. These phyla are likely to be transferred to cheese as they are among the major phyla found in the dairy environment (25, 46). Genera of interest for milk processing were identified in the analysis, but they were present in very low abundance. For example, OTUs corresponding to the lactic acid bacterium *Lactococcus sp*., a genus encompassing species commonly used as cheese starters, were found in 30 samples over 54 (17 samples with more than 100 OTUs). *Brevundimonas, Chryseobacterium, Sphingomonas, Frigoribacterium*, *Pantoea,* and *Rhodococcus*, particularly abundant in the phyllosphere samples, are genera commonly described in dairy environments (47). To study bacterial transfer from the grassland phyllosphere to milk and the dairy environment, further investigations are needed by using culture-dependent and culture-independent methods.

Grassland management parameters (e.g., rate of fertilizer application and cutting frequency) may also influence the phyllosphere microbiota composition and structure as they do in soils (48, 49). In the current study, we chose to sample leaves from two grasslands having the same grazing pressure to only take into account the use or not of N fertilization. In an evaluation of the effect of extensification of grassland management, Behrendt et al. (2004) found that the population density of heterotrophic bacteria in the phyllosphere was higher in fertilized plots. In addition, bacterial colonization was also influenced by cutting frequency, with a lower population density in two-cut management than in one-cut management, but only in unfertilized grassland. In our study, N fertilization had no effect on the total microbial population counts and a weak effect on *Methylobacteria* counts, which were slightly higher in spring and lower in autumn in the fertilized grassland. N fertilization also significantly altered the structure of the phyllosphere microbiota, but the effect was weak compared to the effects of the other studied factors, namely, plant species and sampling period.

### Effect of leaf morphology and biochemistry on microbiota composition

In the current study, the differences in microbiota composition between plant species may be attributed to leaf morphology, as previously observed in spruce species (*Picea* spp.) (11). It could explain the significant differences between the microbiota of *T. repens* and those of the two *Poaceae L. perenne* and *H. lanatus*. Leaf surface topography impacts the retention of water and affects bacterial survival under conditions of low relative humidity, suggesting topography-dependent protection from desiccation (50). Thus, the differences in leaf surface topography observed among the three studied species, namely, bumpy surface with high stomata density in *T. repens*, deep parallel grooves along the leaf veins in *L. perenne,* and trichomes in *H. lanatus*, may contribute to differences in microbiota colonization and composition by modulating water retention patterns at the leaf surface. The large leaf veins of *L. perenne* and numerous trichomes of *H. lanatus* may have led to high microbial colonization due to higher availability of nutrients (50). Contrary to what might be expected, the most colonized leaves were not those of *Poaceae* but those of *T. repens*, which are characterized by a high density of stomata known to enhance bacterial colonization (51).

In addition to sucrose, glucose, and fructose, which are synthesized by all plant species and can influence microbial assemblages (51, 52), specific soluble carbohydrates found in certain plant species may contribute to shape the phyllosphere microbiota. Pinitol is a cyclitol synthesized by a limited number of plant species, including *Pinaceae* and *Fabaceae*, in which it helps tolerate abiotic stresses such as water deficit and high salinity (53). The high abundance of pinitol in *T. repens* leaves may influence phyllosphere microbiota as it can be metabolized by some bacteria like *Erwinia* and *Serratia*, as evidenced in pine trees (54), and also exhibits antifungal activity (55). Indeed, the present study highlights the significant correlation of pinitol level in leaves and the structure of the phyllosphere microbiota of *T. repens*. In addition, the difference in phyllosphere microbial communities between *T. repens* and the two *Poaceae* may also be due to the presence of fructans in *Poaceae*. A wide range of bacteria and fungi can metabolize fructans by the action of extracellular endo- and exo-fructanases (56). In bacteria, fructanases have been identified in genera such as *Bacillus, Actinomyces, Bacteroides, Paenibacillus,* and *Gluconacetobacter* (56). More specifically, it has been shown that a levanase from *Bacillus subtilis* is able to degrade the mixed-linkage fructans from *L. perenne* (57). The presence of fructans in leaves modified the abundance of several bacterial groups of the phyllosphere microbial communities, with bacilli being dominant, while their absence favored *Actinobacteria* (58). In our study, no link between fructan levels and phyllosphere microbiota composition was demonstrated, but it can be hypothesized that their presence in *L. perenne* and *H. lanatus* contributed to the differences between their phyllosphere microbial communities and that of *T. repens*. Experimental validation would be required to reliably demonstrate this effect, possibly using an approach based on previous works (59).

### Influence of short-term and seasonal variations of weather on phyllosphere microbiota

We found that the composition of grassland microbiomes was driven by the period of the year (P1 versus P2 and P3). This result is supported by the study of Copeland et al. (60), which showed that leaf microbiota, which was very strongly influenced by the soil microbiota at the beginning of the season, became significantly less diverse with time, with a greater proportion of leaf-specific taxa shared among all samples. In our study, the weather factors were important drivers of the microbiota composition, especially the TR, the Rf amount, and to a lesser extent, the R and evapotranspiration observed during the days preceding the leaf sampling. This is congruent with studies showing that the responses of phyllosphere microbiota to environmental stresses such as drought (8) or temperature elevation (3) are characterized by an ecological drift. Likewise, our findings are consistent with observations from spinach cultivation (*Spinacia oleracea*), where factors such as wind speed, solar radiation, and relative humidity were shown to influence the bacterial composition on leaf surfaces (61). However, the phyllosphere microbiota of broadleaf cattail (*Typha latifolia*) was found to be unaffected by short-term weather patterns but particularly sensitive to climatic and leaf-associated changes occurring with seasonal progression (62), indicating that the short-term environmental variations should not be overestimated compared to seasonal factors. Stone and Jackson (62) suggested that the abundance of *Sphingomonas* can be a useful indicator for assessing the influence of short-term or seasonal factors as it has been associated with senescent leaves. Accordingly, in the present study, *Sphingomonas* appeared generally more abundant in the phyllosphere of leaves sampled in P2, after a period of low level of Rf with high evapotranspiration, which may have enhanced leaf senescence.

More generally, shifts in the phyllosphere microbiota composition due to climate change have recently been demonstrated in experiments of long-term moderate warming in permanent grassland (3). Interestingly, the variations of raw milk microbiota are also linked to season and geography, especially in milk from pasture-based systems (63), reinforcing the evidence of connections between phyllosphere and raw milk microbiota.

### Conclusion

The foliar microbiome contributes to several ecosystem functions, including nutrient and water cycling, that can be altered by global changes (27). Here, we presented new insights into plant and environmental factors that shape grassland phyllosphere microbiota.

Many reviews on plant phyllosphere microbiota focus on the plant pathogen interactions for plant protection strategies (64–66). Here, we highlighted grasslands as potential reservoirs of beneficial microorganisms for plants and dairy-derived products. Along with other recent studies (8, 9, 21, 22, 67), our study shows that the phyllosphere microbiome must be taken into account when assessing the ecosystem services of grasslands as it can contribute not only to supporting services through the biodiversity it contains but also to provisioning services through the microorganisms that can be useful for the agriculture and food sectors.

## MATERIALS AND METHODS

### Sampling

Samples collection was performed at the INRAE Experimental Unit of Le Pin-au-Haras in the northwestern part of France (Normandy, France, 48°43′27.7” N, 0°10′52.2 E). Two adjacent permanent grasslands, typical of mesophilic grazed grasslands in temperate European regions, were selected: one receiving each year nitrogen fertilization (150 kg N.ha^-1^) and then named F+ and the other not receiving any fertilization (F−). Aside from the fertilization, the two grasslands shared the same characteristics: identical topography, soil composition, cattle grazing management, and climate conditions. In each grassland, three sampling zones (1–3) were defined (see Fig. S5 at https://doi.org/10.57745/MDJY8I). In each zone, leaves were collected from three monocot species, *H. lanatus*, *L. perenne,* and *T. repens*. For each plant species, aerial plant tissues 5 cm above ground were harvested using ethanol-cleaned scissors and wearing gloves. Approximately 30 g of fresh plant tissues was sampled for bacterial analysis and 5 g for biochemical analysis. The samples, mainly composed of leaf blades, were immediately placed in sterile bags and put on ice during transportation. Sampling campaigns took place in 2016 at three periods, P1, P2, and P3, to cover the typical grazing period in temperate European regions (see Table S1 at https://doi.org/10.57745/MDJY8I). The sampling dates result from the possibility of entering the grassland, i.e., between two periods of grazing of the herd of cows, which passed successively from one to the other of the two adjacent grasslands, and considering a delay of at least 1 week of regrowth after grazing. The mean weather parameters were indeed different between periods (see Table S2 at https://doi.org/10.57745/MDJY8I), P1 was characterized by intermediate daily average temperature (15.9°C) and the highest Rf (2.2 mm per day), P2 by the highest daily average temperature (18.8°C) and the lowest Rf (0.9 mm per day) and P3 by the lowest daily average temperature (14.9°C) and intermediate Rf (1.6 mm per day). For each period, the samples were collected at the same lag times after grazing to avoid interference with the plant growth parameter. A total of 54 samples (2 grasslands × 3 zones × 3 plant species × 3 periods) were collected (see Table S2 at https://doi.org/10.57745/MDJY8I).

### Bacterial enumeration

For all the samples, 30 g of leaves was immersed in 270 mL phosphate-buffered saline (PBS) buffer (ThermoFisher) containing 0.1% Tween 20 (ThermoFisher) in sterile stomacher bags. The bags were shaken at 110 rpm for 5 min at room temperature before a sonication step of 30 s with 2-s pulses at 40W and 1-s pauses between pulses. Three milliliters of the obtained sample suspensions was harvested for bacterial enumeration, the remaining of the sample suspension being reserved for DNA extraction (see next section). Decimal dilutions in tryptone salt (Biokar) were prepared. Standard plate counts were enumerated on PCA (Biokar) incubated at 30°C for 72 h and methylobacteria on *Methylobacterium* agar supplemented with 0.5% methanol (68) incubated at 25°C for 72 h. Bacterial counts were expressed as mean values of three replicates in cfu/g.

### DNA extraction and high-throughput sequencing

The sample suspensions were centrifuged at 4,500 rpm for 5 min at 4°C. The pellet was washed once in 10 mL PBS buffer and centrifuged again at 4,500 rpm for 5 min at 4°C. The pellet was stored at −80°C until further processing.

Total DNA was extracted from the pellets using the PowerFood microbial DNA kit (Mobio) with slight modifications from the manufacturer’s instructions for the lysis step. Briefly, the bacterial pellet was resuspended in 450 µL solution PF1 and transferred into a sterile 2 mL microtube containing 0.1 mm diameter zirconium beads. Mechanical lysis was performed using a MM200 mixer mill (Retsch) for 5 min at 25 Hz. The amount and quality of isolated genomic DNA were verified using the QuantiFluor dsDNA System (Promega) and gel electrophoresis. Amplification of the V1–V3 region (~ 500 bases) of the 16S rRNA gene, amplicon library construction using the InView Microbiome Profiling 2.0 service, and Illumina MiSeq paired-end sequencing (2 × 300 pb) were performed at GATC Biotech (Konstanz, Germany).

### Bioinformatic analysis of the sequencing data

Sequence analysis was performed using the Galaxy-supported pipeline FROGS (69). Briefly, the clustering used Swarm, the chimera removal used VSEARCH, combined with original cross-sample validation, and filtering was performed by keeping OTU when present in at least three samples and with a minimum relative abundance of 0.0005. Statistical analysis was performed using the Phyloseq R package implemented in FROGS (FROGSSTAT), with Bray-Curtis distance to obtain dissimilarity matrices. To identify genera that may be driving the significant differences detected between plant species, differential abundance analysis was determined on raw genus-level count data, using DESeq2 implemented in FROGS.

### SEM

Leaf sections were fixed with 1% glutaraldehyde in 0.1M phosphate buffer pH 7.0 for 1 week at 4°C, with naturally floating samples being kept immersed in the fixative liquid with a special tube. Samples were rinsed in 0.1M phosphate buffer pH 7.0 and then dehydrated to critical point in 70%–100% progressive ethanol bath (CPD 030 LEICA Microsystem). The cells were pulverized with platinum and observed under a JEOL 6400F SEM (JEOL, Croissy sur Seine, France).

### Biochemical analyses

Plant tissues were freeze-dried and ball-milled ground to a fine powder before analysis. WSC was extracted from plant tissue powder through three successive aqueous extractions for 15 min at 80°C, 60°C, and 60°C. After each extraction, the samples were centrifuged at 10,000 × *g* for 10 min. The supernatant was kept, and pure water was added to the pellet for the following extraction. The three successive supernatants were pooled, freeze-dried, and the residue was dissolved in pure water (0.5 mL for 50 mg of leaf powder). To remove charged compounds before high-performance liquid chromatography (HPLC), aliquots of the extract (100 μL) were passed through minicolumns (Mobicols from MoBiTec, Göttingen, Germany) packed, from bottom to top, with 150 μL of Dowex 1×8 200–400 mesh (Sigma-Aldrich, St. Louis, USA), 80 μL of polyvinylpolypyrrolidone (Sigma-Aldrich), and 250 μL of Dowex 50W X8-400 X8–400 H^+^-form (Sigma-Aldrich). Soluble carbohydrates were separated and quantified by HPLC on a cation exchange column (Sugar-PAK, 300 × 6.5 mm, Millipore Waters, Milford, MA) eluted at 0.5 mL min^−1^ and 85°C with 0.1 mM Ca-EDTA in water with a refractometer as the sugar detector. For *H. lanatus* and *L. perenne* samples, sucrose, glucose, and fructose were quantified by comparison with sucrose, glucose, and fructose standards, respectively. For *T. repens*, raffinose, sucrose, and fructose were quantified by comparison with raffinose, sucrose, and fructose standards, respectively. In *T. repens*, since glucose and pinitol have the same retention time in the Sugar-PAK column, glucose concentration was determined with the D-Fructose/D-Glucose Assay Kit (Megazyme International, County Wicklow, Ireland). Pinitol concentration was then determined by the difference between the concentration of the sum of glucose and pinitol determined following HPLC and the glucose concentration determined with the enzymatic kit. In *H. lanatus* and *L. perenne*, fructans were analyzed with the fructan HK assay kit (Megazyme International) with an adapted procedure. Briefly, 20 µL aliquots of extract was incubated in a 1.5 mL tube with 20 µL of sucrose-maltase solution at 40°C for 30 min. After disaccharide hydrolysis, 50 µL of pH 4.5, 100 mM sodium acetate buffer was added to each microtube. Aliquots (15 µL) of this solution were deposited at the bottom of a well in a microplate and incubated with 7.5 µL of 100 mM sodium acetate buffer with or without fructanase at 40°C for 30 min to, respectively, complete hydrolysis or not of fructan to fructose and glucose. Measurements of fructose and glucose released were performed by incubating the hydrolysate with 150 µL of water, 15 µL of pH 7.6 buffer (buffer 1 of the fructan HK assay kit, Megazyme International), 7.5 µL of NADP^+^/ATP solution, and 2 µL of hexokinase/phosphoglucose-isomerase/glucose-6-phosphate dehydrogenase solution at room temperature for 12 min. Fructan concentrations were calculated with the measurement of absorbance at 340 nm before and after incubation.

Leaf C and N concentrations (in mass per dry mass) and δ^13^C (39) were measured with an IsoPrime mass spectrometer (Elementar, Lyon, France) connected to an elemental analyzer (EA3000, Euro Vector, Milan, Italy).

### Collection of weather data

Weather data were monitored from the INRAE CLIMATIK platform (https://agroclim.inrae.fr/climatik/, in French) managed by the AgroClim laboratory of Avignon, France (70). The weather station used was located in the non-fertilized grassland (F−) (AgroClim station number 61328003). Daily weather parameters were taken over a 30-day period before each sampling date. The average values of each parameter over a period of 1, 3, 15, and 30 days before each sampling date were then calculated, and their significance on bacterial microbiota composition was evaluated in an NMDS model using R software (v. 3.5.4) as previously described (10). This allowed the selection of the most significant weather variables, namely, Rf, TR, ETo, R, and W, over a 3- and a 15-day period before each sampling date (see Table S3 at https://doi.org/10.57745/MDJY8I).

### Statistical analysis

The effects of sampling period, fertilization level, and plant species on leaf biochemical parameters were analyzed by three-way ANOVA. Prior to ANOVA, a Shapiro-Wilk test (95%) and a Bartlett test (95%) were performed on each set of data to assess data normality and homogeneity of variances, respectively. Inside each period and fertilization level, the effects of plant species were analyzed by one-way ANOVA. When the effect was significant (*P* ≤ 0.05), the ANOVA was followed by a pairwise comparison (Tukey’s test) to compare the three plant species (*P* ≤ 0.05).

## Data Availability

Sequence reads are available in the BioSample NCBI database under accession numbers SAMN09280971 to SAMN09281024 and in the SRA database under accession number SRP149327.
